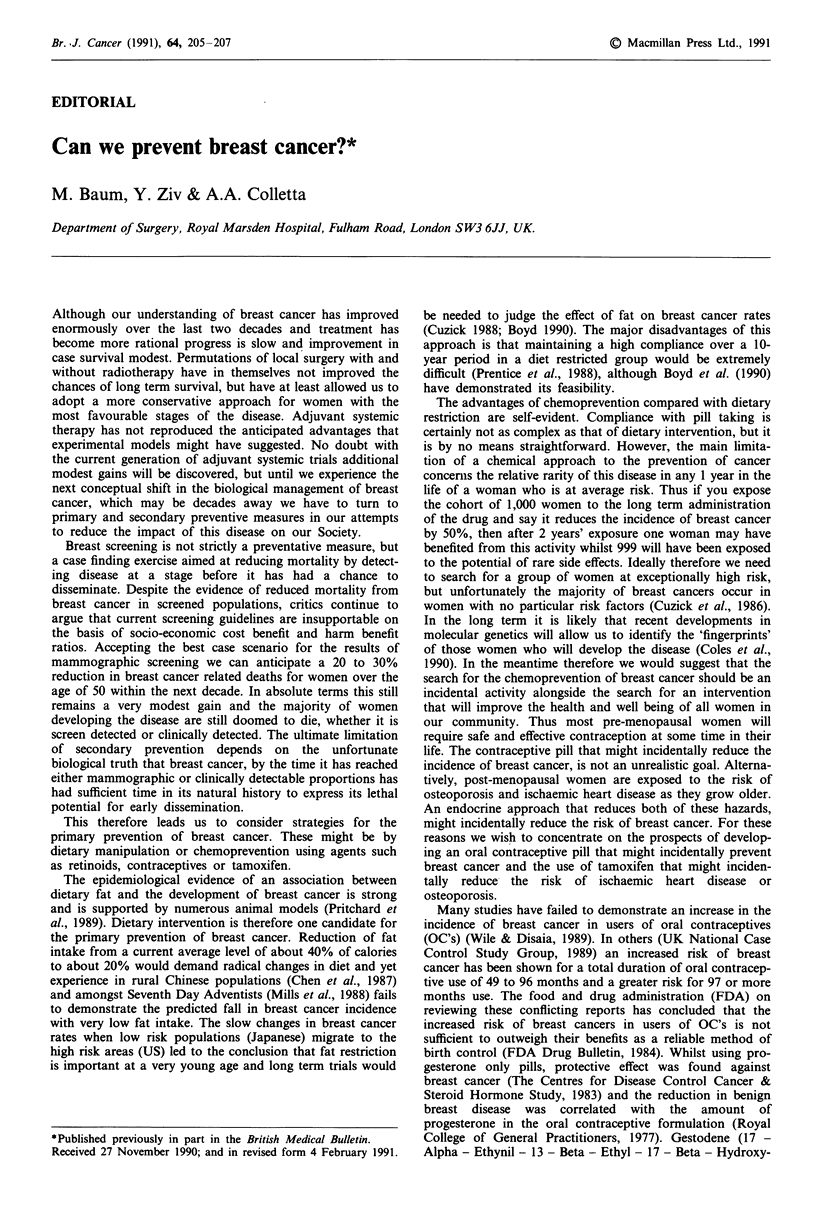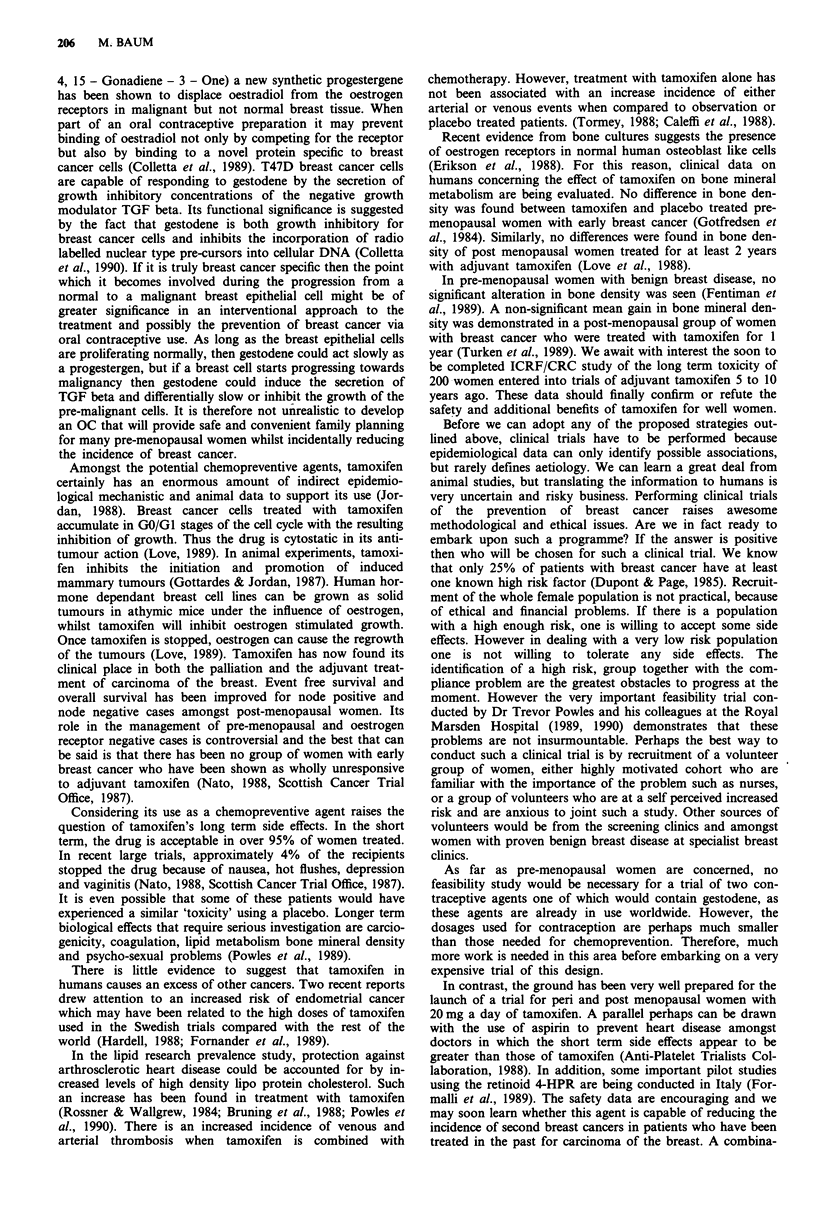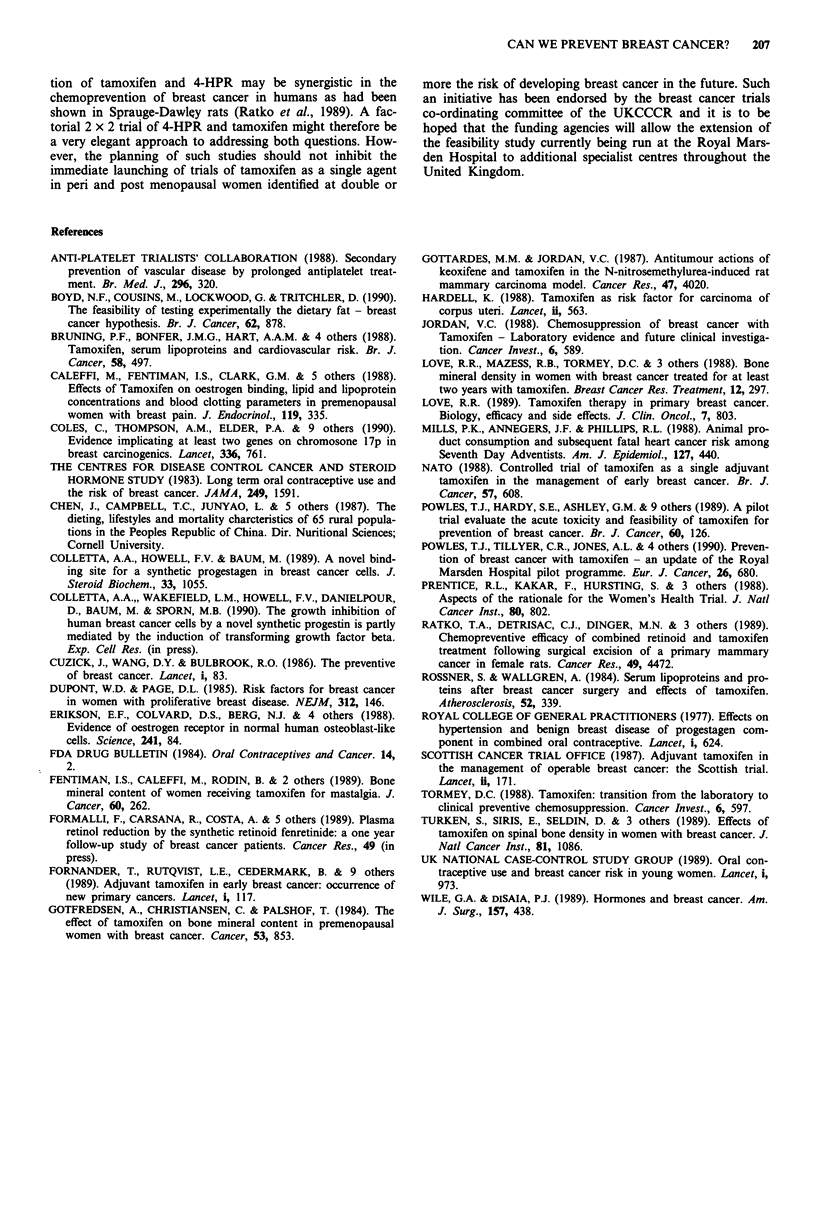# Can we prevent breast cancer?

**DOI:** 10.1038/bjc.1991.276

**Published:** 1991-08

**Authors:** M. Baum, Y. Ziv, A. A. Colletta


Br. J. Cancer (1991), 64, 205–207 © Macmillan Press Ltd., 1991

EDITORIAL

### Can we prevent breast cancer?*

M. Baum, Y. Ziv & A.A. Colletta

*Department of Surgery, Royal Marsden Hospital, Fulham Road, London SW3 6JJ, UK.*

Although our understanding of breast cancer has improved enormously over the last two decades and treatment has become more rational progress is slow and improvement in case survival modest. Permutations of local surgery with and without radiotherapy have in themselves not improved the chances of long term survival, but have at least allowed us to adopt a more conservative approach for women with the most favourable stages of the disease. Adjuvant systemic therapy has not reproduced the anticipated advantages that experimental models might have suggested. No doubt with the current generation of adjuvant systemic trials additional modest gains will be discovered, but until we experience the next conceptual shift in the biological management of breast cancer, which may be decades away we have to turn to primary and secondary preventive measures in our attempts to reduce the impact of this disease on our Society.

Breast screening is not strictly a preventative measure, but a case finding exercise aimed at reducing mortality by detecting disease at a stage before it has had a chance to disseminate. Despite the evidence of reduced mortality from breast cancer in screened populations, critics continue to argue that current screening guidelines are insupportable on the basis of socio-economic cost benefit and harm benefit ratios. Accepting the best case scenario for the results of mammographic screening we can anticipate a 20 to 30% reduction in breast cancer related deaths for women over the age of 50 within the next decade. In absolute terms this still remains a very modest gain and the majority of women developing the disease are still doomed to die, whether it is screen detected or clinically detected. The ultimate limitation of secondary prevention depends on the unfortunate biological truth that breast cancer, by the time it has reached either mammographic or clinically detectable proportions has had sufficient time in its natural history to express its lethal potential for early dissemination.

This therefore leads us to consider strategies for the primary prevention of breast cancer. These might be by dietary manipulation or chemoprevention using agents such as retinoids, contraceptives or tamoxifen.

The epidemiological evidence of an association between dietary fat and the development of breast cancer is strong and is supported by numerous animal models (Pritchard *et al.*, 1989). Dietary intervention is therefore one candidate for the primary prevention of breast cancer. Reduction of fat intake from a current average level of about 40% of calories to about 20% would demand radical changes in diet and yet experience in rural Chinese populations (Chen *et al.*, 1987) and amongst Seventh Day Adventists (Mills *et al.*, 1988) fails to demonstrate the predicted fall in breast cancer incidence with very low fat intake. The slow changes in breast cancer rates when low risk populations (Japanese) migrate to the high risk areas (US) led to the conclusion that fat restriction is important at a very young age and long term trials would be needed to judge the effect of fat on breast cancer rates (Cuzick 1988; Boyd 1990). The major disadvantages of this approach is that maintaining a high compliance over a 10-year period in a diet restricted group would be extremely difficult (Prentice *et al.*, 1988), although Boyd *et al.* (1990) have demonstrated its feasibility.

The advantages of chemoprevention compared with dietary restriction are self-evident. Compliance with pill taking is certainly not as complex as that of dietary intervention, but it is by no means straightforward. However, the main limitation of a chemical approach to the prevention of cancer concerns the relative rarity of this disease in any 1 year in the life of a woman who is at average risk. Thus if you expose the cohort of 1,000 women to the long term administration of the drug and say it reduces the incidence of breast cancer by 50%, then after 2 years' exposure one woman may have benefited from this activity whilst 999 will have been exposed to the potential of rare side effects. Ideally therefore we need to search for a group of women at exceptionally high risk, but unfortunately the majority of breast cancers occur in women with no particular risk factors (Cuzick *et al.*, 1986). In the long term it is likely that recent developments in molecular genetics will allow us to identify the 'fingerprints' of those women who will develop the disease (Coles *et al.*, 1990). In the meantime therefore we would suggest that the search for the chemoprevention of breast cancer should be an incidental activity alongside the search for an intervention that will improve the health and well being of all women in our community. Thus most pre-menopausal women will require safe and effective contraception at some time in their life. The contraceptive pill that might incidentally reduce the incidence of breast cancer, is not an unrealistic goal. Alternatively, post-menopausal women are exposed to the risk of osteoporosis and ischaemic heart disease as they grow older. An endocrine approach that reduces both of these hazards, might incidentally reduce the risk of breast cancer. For these reasons we wish to concentrate on the prospects of developing an oral contraceptive pill that might incidentally prevent breast cancer and the use of tamoxifen that might incidentally reduce the risk of ischaemic heart disease or osteoporosis.

Many studies have failed to demonstrate an increase in the incidence of breast cancer in users of oral contraceptives (OC's) (Wile & Disaia, 1989). In others (UK National Case Control Study Group, 1989) an increased risk of breast cancer has been shown for a total duration of oral contraceptive use of 49 to 96 months and a greater risk for 97 or more months use. The food and drug administration (FDA) on reviewing these conflicting reports has concluded that the increased risk of breast cancers in users of OC's is not sufficient to outweigh their benefits as a reliable method of birth control (FDA Drug Bulletin, 1984). Whilst using progesterone only pills, protective effect was found against breast cancer (The Centres for Disease Control Cancer & Steroid Hormone Study, 1983) and the reduction in benign breast disease was correlated with the amount of progesterone in the oral contraceptive formulation (Royal College of General Practitioners, 1977). Gestodene (17 – Alpha – Ethynil – 13 – Beta – Ethyl – 17 – Beta – Hydroxy-

*Published previously in part in the *British Medical Bulletin*.
Received 27 November 1990; and in revised form 4 February 1991.

4, 15 – Gonadiene – 3 – One) a new synthetic progestergene has been shown to displace oestradiol from the oestrogen receptors in malignant but not normal breast tissue. When part of an oral contraceptive preparation it may prevent binding of oestradiol not only by competing for the receptor but also by binding to a novel protein specific to breast cancer cells (Colletta *et al.*, 1989). T47D breast cancer cells are capable of responding to gestodene by the secretion of growth inhibitory concentrations of the negative growth modulator TGF beta. Its functional significance is suggested by the fact that gestodene is both growth inhibitory for breast cancer cells and inhibits the incorporation of radio labelled nuclear type pre-cursors into cellular DNA (Colletta *et al.*, 1990). If it is truly breast cancer specific then the point which it becomes involved during the progression from a normal to a malignant breast epithelial cell might be of greater significance in an interventional approach to the treatment and possibly the prevention of breast cancer via oral contraceptive use. As long as the breast epithelial cells are proliferating normally, then gestodene could act slowly as a progestergen, but if a breast cell starts progressing towards malignancy then gestodene could induce the secretion of TGF beta and differentially slow or inhibit the growth of the pre-malignant cells. It is therefore not unrealistic to develop an OC that will provide safe and convenient family planning for many pre-menopausal women whilst incidentally reducing the incidence of breast cancer.

Amongst the potential chemopreventive agents, tamoxifen certainly has an enormous amount of indirect epidemiological mechanistic and animal data to support its use (Jordan, 1988). Breast cancer cells treated with tamoxifen accumulate in G0/G1 stages of the cell cycle with the resulting inhibition of growth. Thus the drug is cytostatic in its anti-tumour action (Love, 1989). In animal experiments, tamoxifen inhibits the initiation and promotion of induced mammary tumours (Gottardes & Jordan, 1987). Human hormone dependant breast cell lines can be grown as solid tumours in athymic mice under the influence of oestrogen, whilst tamoxifen will inhibit oestrogen stimulated growth. Once tamoxifen is stopped, oestrogen can cause the regrowth of the tumours (Love, 1989). Tamoxifen has now found its clinical place in both the palliation and the adjuvant treatment of carcinoma of the breast. Event free survival and overall survival has been improved for node positive and node negative cases amongst post-menopausal women. Its role in the management of pre-menopausal and oestrogen receptor negative cases is controversial and the best that can be said is that there has been no group of women with early breast cancer who have been shown as wholly unresponsive to adjuvant tamoxifen (Nato, 1988, Scottish Cancer Trial Office, 1987).

Considering its use as a chemopreventive agent raises the question of tamoxifen's long term side effects. In the short term, the drug is acceptable in over 95% of women treated. In recent large trials, approximately 4% of the recipients stopped the drug because of nausea, hot flushes, depression and vaginitis (Nato, 1988, Scottish Cancer Trial Office, 1987). It is even possible that some of these patients would have experienced a similar 'toxicity' using a placebo. Longer term biological effects that require serious investigation are carciogenicity, coagulation, lipid metabolism bone mineral density and psycho-sexual problems (Powles *et al.*, 1989).

There is little evidence to suggest that tamoxifen in humans causes an excess of other cancers. Two recent reports drew attention to an increased risk of endometrial cancer which may have been related to the high doses of tamoxifen used in the Swedish trials compared with the rest of the world (Hardell, 1988; Fornander *et al.*, 1989).

In the lipid research prevalence study, protection against arthrosclerotic heart disease could be accounted for by increased levels of high density lipo protein cholesterol. Such an increase has been found in treatment with tamoxifen (Rossner & Wallgrew, 1984; Bruning *et al.*, 1988; Powles *et al.*, 1990). There is an increased incidence of venous and arterial thrombosis when tamoxifen is combined with chemotherapy. However, treatment with tamoxifen alone has not been associated with an increase incidence of either arterial or venous events when compared to observation or placebo treated patients. (Tormey, 1988; Caleffi *et al.*, 1988).

Recent evidence from bone cultures suggests the presence of oestrogen receptors in normal human osteoblast like cells (Erikson *et al.*, 1988). For this reason, clinical data on humans concerning the effect of tamoxifen on bone mineral metabolism are being evaluated. No difference in bone density was found between tamoxifen and placebo treated pre-menopausal women with early breast cancer (Gotfredsen *et al.*, 1984). Similarly, no differences were found in bone density of post menopausal women treated for at least 2 years with adjuvant tamoxifen (Love *et al.*, 1988).

In pre-menopausal women with benign breast disease, no significant alteration in bone density was seen (Fentiman *et al.*, 1989). A non-significant mean gain in bone mineral density was demonstrated in a post-menopausal group of women with breast cancer who were treated with tamoxifen for 1 year (Turken *et al.*, 1989). We await with interest the soon to be completed ICRF/CRC study of the long term toxicity of 200 women entered into trials of adjuvant tamoxifen 5 to 10 years ago. These data should finally confirm or refute the safety and additional benefits of tamoxifen for well women.

Before we can adopt any of the proposed strategies outlined above, clinical trials have to be performed because epidemiological data can only identify possible associations, but rarely defines aetiology. We can learn a great deal from animal studies, but translating the information to humans is very uncertain and risky business. Performing clinical trials of the prevention of breast cancer raises awesome methodological and ethical issues. Are we in fact ready to embark upon such a programme? If the answer is positive then who will be chosen for such a clinical trial. We know that only 25% of patients with breast cancer have at least one known high risk factor (Dupont & Page, 1985). Recruitment of the whole female population is not practical, because of ethical and financial problems. If there is a population with a high enough risk, one is willing to accept some side effects. However in dealing with a very low risk population one is not willing to tolerate any side effects. The identification of a high risk, group together with the compliance problem are the greatest obstacles to progress at the moment. However the very important feasibility trial conducted by Dr Trevor Powles and his colleagues at the Royal Marsden Hospital (1989, 1990) demonstrates that these problems are not insurmountable. Perhaps the best way to conduct such a clinical trial is by recruitment of a volunteer group of women, either highly motivated cohort who are familiar with the importance of the problem such as nurses, or a group of volunteers who are at a self perceived increased risk and are anxious to joint such a study. Other sources of volunteers would be from the screening clinics and amongst women with proven benign breast disease at specialist breast clinics.

As far as pre-menopausal women are concerned, no feasibility study would be necessary for a trial of two contraceptive agents one of which would contain gestodene, as these agents are already in use worldwide. However, the dosages used for contraception are perhaps much smaller than those needed for chemoprevention. Therefore, much more work is needed in this area before embarking on a very expensive trial of this design.

In contrast, the ground has been very well prepared for the launch of a trial for peri and post menopausal women with 20 mg a day of tamoxifen. A parallel perhaps can be drawn with the use of aspirin to prevent heart disease amongst doctors in which the short term side effects appear to be greater than those of tamoxifen (Anti-Platelet Trialists Collaboration, 1988). In addition, some important pilot studies using the retinoid 4-HPR are being conducted in Italy (Formalli *et al.*, 1989). The safety data are encouraging and we may soon learn whether this agent is capable of reducing the incidence of second breast cancers in patients who have been treated in the past for carcinoma of the breast. A combination of tamoxifen and 4-HPR may be synergistic in the chemoprevention of breast cancer in humans as had been shown in Sprauge-Dawley rats (Ratko *et al.*, 1989). A factorial 2 × 2 trial of 4-HPR and tamoxifen might therefore be a very elegant approach to addressing both questions. However, the planning of such studies should not inhibit the immediate launching of trials of tamoxifen as a single agent in peri and post menopausal women identified at double or more the risk of developing breast cancer in the future. Such an initiative has been endorsed by the breast cancer trials co-ordinating committee of the UKCCCR and it is to be hoped that the funding agencies will allow the extension of the feasibility study currently being run at the Royal Marsden Hospital to additional specialist centres throughout the United Kingdom.

### References

ANTI-PLATELET TRIALISTS' COLLABORATION (1988). Secondary prevention of vascular disease by prolonged antiplatelet treatment. *Br. Med. J.*, **296**, 320.

BOYD, N.F., COUSINS, M., LOCKWOOD, G. & TRITCHLER, D. (1990). The feasibility of testing experimentally the dietary fat – breast cancer hypothesis. *Br. J. Cancer*, **62**, 878.

BRUNING, P.F., BONFER, J.M.G., HART, A.A.M. & 4 others (1988). Tamoxifen, serum lipoproteins and cardiovascular risk. *Br. J. Cancer*, **58**, 497.

CALEFFI, M., FENTIMAN, I.S., CLARK, G.M. & 5 others (1988). Effects of Tamoxifen on oestrogen binding, lipid and lipoprotein concentrations and blood clotting parameters in premenopausal women with breast pain. *J. Endocrinol.*, **119**, 335.

COLES, C., THOMPSON, A.M., ELDER, P.A. & 9 others (1990). Evidence implicating at least two genes on chromosone 17p in breast carcinogenics. *Lancet*, **336**, 761.

THE CENTRES FOR DISEASE CONTROL CANCER AND STEROID HORMONE STUDY (1983). Long term oral contraceptive use and the risk of breast cancer. *JAMA*, **249**, 1591.

CHEN, J., CAMPBELL, T.C., JUNYAO, L. & 5 others (1987). The dieting, lifestyles and mortality charcteristics of 65 rural populations in the Peoples Republic of China. Dir. Nuritional Sciences; Cornell University.

COLLETTA, A.A., HOWELL, F.V. & BAUM, M. (1989). A novel binding site for a synthetic progestagen in breast cancer cells. *J. Steroid Biochem.*, **33**, 1055.

COLLETTA, A.A.,, WAKEFIELD, L.M., HOWELL, F.V., DANIELPOUR, D., BAUM, M. & SPORN, M.B. (1990). The growth inhibition of human breast cancer cells by a novel synthetic progestin is partly mediated by the induction of transforming growth factor beta. *Exp. Cell Res.* (in press).

CUZICK, J., WANG, D.Y. & BULBROOK, R.O. (1986). The preventive of breast cancer. *Lancet*, **i**, 83.

DUPONT, W.D. & PAGE, D.L. (1985). Risk factors for breast cancer in women with proliferative breast disease. *NEJM*, **312**, 146.

ERIKSON, E.F., COLVARD, D.S., BERG, N.J. & 4 others (1988). Evidence of oestrogen receptor in normal human osteoblast-like cells. *Science*, **241**, 84.

FDA DRUG BULLETIN (1984). *Oral Contraceptives and Cancer.* **14**, 2.

FENTIMAN, I.S., CALEFFI, M., RODIN, B. & 2 others (1989). Bone mineral content of women receiving tamoxifen for mastalgia. *J. Cancer*, **60**, 262.

FORMALLI, F., CARSANA, R., COSTA, A. & 5 others (1989). Plasma retinol reduction by the synthetic retinoid fenretinide: a one year follow-up study of breast cancer patients. *Cancer Res.*, **49** (in press).

FORNANDER, T., RUTQVIST, L.E., CEDERMARK, B. & 9 others (1989). Adjuvant tamoxifen in early breast cancer: occurrence of new primary cancers. *Lancet*, **i**, 117.

GOTFREDSEN, A., CHRISTIANSEN, C. & PALSHOF, T. (1984). The effect of tamoxifen on bone mineral content in premenopausal women with breast cancer. *Cancer*, **53**, 853.

GOTTARDES, M.M. & JORDAN, V.C. (1987). Antitumour actions of keoxifene and tamoxifen in the N-nitrosemethylurea-induced rat mammary carcinoma model. *Cancer Res.*, **47**, 4020.

HARDELL, K. (1988). Tamoxifen as risk factor for carcinoma of corpus uteri. *Lancet*, **ii**, 563.

JORDAN, V.C. (1988). Chemosuppression of breast cancer with Tamoxifen – Laboratory evidence and future clinical investigation. *Cancer Invest.*, **6**, 589.

LOVE, R.R., MAZESS, R.B., TORMEY, D.C. & 3 others (1988). Bone mineral density in women with breast cancer treated for at least two years with tamoxifen. *Breast Cancer Res. Treatment*, **12**, 297.

LOVE, R.R. (1989). Tamoxifen therapy in primary breast cancer. Biology, efficacy and side effects. *J. Clin. Oncol.*, **7**, 803.

MILLS, P.K., ANNEGERS, J.F. & PHILLIPS, R.L. (1988). Animal product consumption and subsequent fatal heart cancer risk among Seventh Day Adventists. *Am. J. Epidemiol.*, **127**, 440.

NATO (1988). Controlled trial of tamoxifen as a single adjuvant tamoxifen in the management of early breast cancer. *Br. J. Cancer*, **57**, 608.

POWLES, T.J., HARDY, S.E., ASHLEY, G.M. & 9 others (1989). A pilot trial evaluate the acute toxicity and feasibility of tamoxifen for prevention of breast cancer. *Br. J. Cancer*, **60**, 126.

POWLES, T.J., TILLYER, C.R., JONES, A.L. & 4 others (1990). Prevention of breast cancer with tamoxifen – an update of the Royal Marsden Hospital pilot programme. *Eur. J. Cancer*, **26**, 680.

PRENTICE, R.L., KAKAR, F., HURSTING, S. & 3 others (1988). Aspects of the rationale for the Women's Health Trial. *J. Natl Cancer Inst.*, **80**, 802.

RATKO, T.A., DETRISAC, C.J., DINGER, M.N. & 3 others (1989). Chemopreventive efficacy of combined retinoid and tamoxifen treatment following surgical excision of a primary mammary cancer in female rats. *Cancer Res.*, **49**, 4472.

ROSSNER, S. & WALLGREN, A. (1984). Serum lipoproteins and proteins after breast cancer surgery and effects of tamoxifen. *Atherosclerosis*, **52**, 339.

ROYAL COLLEGE OF GENERAL PRACTITIONERS (1977). Effects on hypertension and benign breast disease of progestagen component in combined oral contraceptive. *Lancet*, **i**, 624.

SCOTTISH CANCER TRIAL OFFICE (1987). Adjuvant tamoxifen in the management of operable breast cancer: the Scottish trial. *Lancet*, **ii**, 171.

TORMEY, D.C. (1988). Tamoxifen: transition from the laboratory to clinical preventive chemosuppression. *Cancer Invest.*, **6**, 597.

TURKEN, S., SIRIS, E., SELDIN, D. & 3 others (1989). Effects of tamoxifen on spinal bone density in women with breast cancer. *J. Natl Cancer Inst.*, **81**, 1086.

UK NATIONAL CASE-CONTROL STUDY GROUP (1989). Oral contraceptive use and breast cancer risk in young women. *Lancet*, **i**, 973.

WILE, G.A. & DISAIA, P.J. (1989). Hormones and breast cancer. *Am. J. Surg.*, **157**, 438.